# Cross-Regulation of Metabolic and Immune Pathways in Plants Under Hypoxic Conditions

**DOI:** 10.3390/plants15071029

**Published:** 2026-03-27

**Authors:** Javier-David Vega-Arroy, Miguel Plascencia-Espinosa

**Affiliations:** Centro de Investigación en Biotecnología Aplicada del Instituto Politécnico Nacional (CIBA-IPN), Ex-Hacienda San Juan Molino Carretera Estatal, Km 1.5, Santa Inés Tecuexcomac, Tlaxcala 90700, Mexico

**Keywords:** Hypoxia, anoxia, normoxia, reoxygenation, differential expression, promoter, transcription factor, cell signaling

## Abstract

Plants are sessile organisms that use molecular oxygen to perform basic metabolic functions. However, when oxygen availability decreases to 1–5% (hypoxic stress), the plant responds transcriptionally to adjust its metabolism and survive the stress. It has been observed that during hypoxia, adenosine triphosphate (ATP) levels decrease drastically, which could trigger plant death. However, despite experiencing an energy deficit, it has been observed that during hypoxia, plants induce defense mechanisms against pathogens. Plants manage to evade pathogenic microorganisms during an energy deficit by using complex signaling networks and different levels of regulation (transcriptional, post-translational, physiological, metabolomic, etc.) that converge to respond to both types of stress (biotic and abiotic). Understanding this phenomenon would have potential applications for agriculture and crop improvement. Therefore, this review details the main mechanisms of plant response to hypoxia and how this affects immunity mechanisms, highlighting the participation of ERF-VII transcription factors as oxygen sensors and their ability to bind to the GCC-box present in promoter regions of defense genes, MAPK signaling pathways, hormonal pathways, ROS, and Ca^2+^.

## 1. Introduction

Plants are sessile, aerobic organisms that use oxygen (O_2_) to perform their metabolic functions, primarily for the oxidation of organic molecules to produce energy in the form of ATP. Depending on the O_2_ concentration available to the plant, the condition can be classified as anoxia (complete absence of O_2_) or hypoxia (when the concentration is between 1 and 5%) [1,2]. When the O_2_ available to the plant decreases, it readjusts its metabolism to adapt to hypoxic stress, characterized by low ATP production [2,3]. In nature, plants are exposed to both acute and chronic hypoxia. Chronic hypoxia is a non-stressful physiological condition in which specific cells or tissues are exposed to low-oxygen concentrations. In contrast, acute hypoxia is triggered by external factors such as flooding and waterlogging. Additionally, some studies have observed an induced hypoxic state in plants growing at high altitudes [2,4,5]. When oxygen availability is restored and hypoxic stress ends, the plant enters a state of reoxygenation. During this phase, the plant attempts to recover its baseline phenotype, undergoing biochemical and metabolic changes such as the generation of reactive oxygen species (ROS) and free radicals, changes in mitochondrial functions, photoinhibition, among others [3,6,7,8,9]. In some cases, these metabolic changes occur so abruptly that they lead to plant death. For instance, the generation of free radicals can cause oxidative damage to proteins, lipids, and DNA (deoxyribonucleic acid) [8,9]. Nevertheless, plants try to adapt to the stress caused by reoxygenation; for example, Garnczarska et al. [6] observed an antioxidant defense mechanism in the roots of lupin seedlings (*Lupinus luteus* L.) during reoxygenation, mediated by enzymes that scavenged free radicals. Collectively, these findings demonstrate that plant responses to oxygen availability whether anoxia or hypoxia and subsequent reoxygenation represent a dynamic temporal phenomenon. Throughout this process, plants continuously readjust their gene expression to adapt; failure to do so effectively may culminate in cell death [6].

A relatively understudied aspect of plant hypoxia responses is how this physiological state may prime plants to better withstand pathogen infections. Emerging evidence suggests that the transcriptomic and metabolic reprogramming triggered by hypoxia is interconnected with immune signaling pathways either through (1) compensatory physiological mechanisms or (2) a coordinated response [10,11].

This review focuses on plant response to acute hypoxia and how its underlying physiological and molecular changes interconnect with plant defense mechanisms against pathogens.

## 2. Linking Hypoxia Signaling to Plant Immunity: Molecular Mechanisms and Defensive Adaptations

Plants respond to hypoxia through a highly coordinated network of molecular, transcriptional, and physiological mechanisms that allow them to perceive reduced oxygen levels and adjust their metabolism, while simultaneously activating defense pathways. At the heart of this response are the ERF-VII’s (group RAP and Hypoxia Responsive ERF or HRE) transcription factors, which stabilize and translocate to the cell nucleus in the absence of oxygen to induce genes whose expression mitigates hypoxic stress while regulating genes related to plant immunity through their interaction with the GCC-box promoter sequence. In parallel, plants deploy compensatory regulatory mechanisms and multiple signaling pathways—including hormonal signals, MAPK, ROS, and Ca^2+^—that integrate the perception of hypoxia with the immune response against pathogens. These molecular events are accompanied by structural and physiological adjustments that either enhance or impair plant survival depending on the system’s conditions. The following sections provide an overview of the main components of this integrated response, from oxygen detection to activation of defenses and physiological remodeling.

### 2.1. Molecular Mechanism of Hypoxia Detection in Plants: Interaction Between PCO and RF-VII Proteins

**Figure 1:**
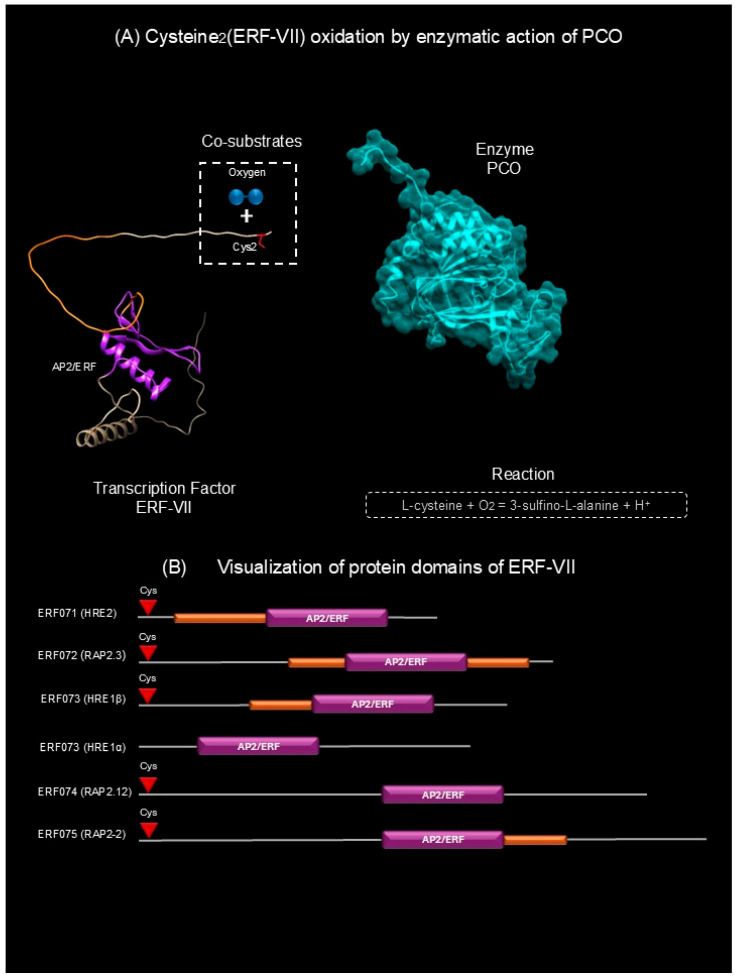
Interaction between ERF-VII and PCO enzyme. (**A**) Cysteine2 (ERF-VII) oxidation by enzymatic action of PCO. (**B**) Visualization of protein domains of ERF-VII (ribbon representation). The red color represents the N-terminal Cys; orange color represents disordered regions, and the purple color represents the DNA-binding domain. This figure was created using UCSF Chimera 1.15 and Microsoft 365 (Office packages).

Plants sense and respond to O_2_ concentration through the interaction of two key proteins: members of family VII of ethylene-responsive transcription factors (ERF-VIIs) and plant protein oxidase (PCO) enzymes (Figure 1A) [4,12,13]. ERF-VIIs are characterized by two functional domains: the AP2/ERF (APETALA2/ethylene-responsive factor) domain, with DNA binding capacity and an N-terminal cysteine residue (Cys2) (Figure 1B), that serves as a co-substrate for PCOs. PCOs, in turn, exhibit cysteine di-oxygenase activity, using both ERF-VII and O_2_ as co-substrates, obeying the following reaction (Figure 1A) (Equation (1)) [4,14].

L-cysteine + O_2_ = 3-sulfino-L-alanine + H^+^ (1)

Equation (1). Reaction catalyzed by PCO proteins which use the N-terminal cysteine (Cys2) present in ERF-VIIs and molecular oxygen as substrates to generate cis-sulfinic acid.

**Figure 2:**
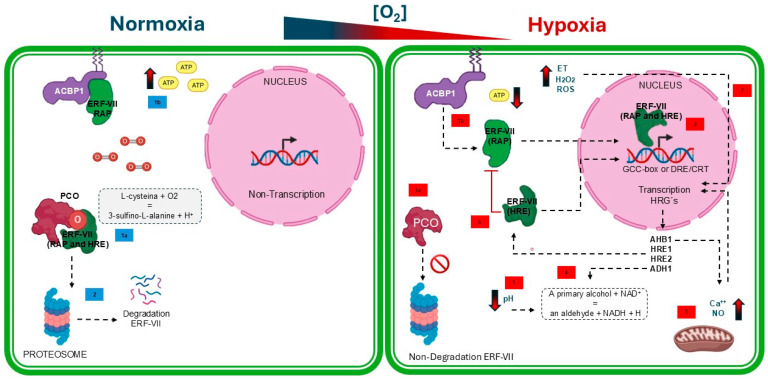
Molecular mechanism of hypoxia detection in plants. Under normoxic conditions, (1a blue) PCO proteins oxygenate ERF-VII transcription factors, (1b blue) ERF-VII (RAP group) remains anchored to ACBP1 proteins due to the high concentration of ATP and (2 blue) consequently target them to the proteasome for degradation, besides the ERF-VII (group RAP) remain anchored to the membrane by interaction with ACBP1 protein. Under hypoxic conditions, oxygen acts as the limiting reagent, so (1a red) ERF-VII are not oxygenated and consequently, (1b red) ERF-VII (RAP) dissociate from ACBP1 proteins due to decreased ATP (2 red) translocate to the nucleus where they induce hypoxia-responsive genes (3 red) It increases calcium flow and NO signaling, (4 red) fermentative pathways are induced and (5 red) cytosolic pH decreases, (6 red) The positive induction of HRE (Hypoxia Responsive ERF) negatively regulates RAP, (7 red) ET, ROS, and H_2_O_2_ signaling occurs during hypoxia. Color blue represents molecular mechanisms under normoxic conditions; color red represents molecular mechanisms under hypoxic conditions. Dotted black arrows represent induction mechanisms; solid red lines represent inhibition mechanisms. The thick arrows with a color gradient from blue to red indicate a decrease in concentration. This figure was created using BioRender Vega-Arroy & Plascencia-Espinosa (2026) https://app.biorender.com/illustrations/69c49a65941dc56988070df9?slideId=30393b71-2300-423c-afca-064caf336dc4 and Microsoft 365 (Office packages).

Under normoxic conditions (21% O_2_), the N-terminal cysteine residue of ERF-VII undergoes continuous oxidation by PCOs. This oxidation generates cis-sulfinic acid targeting ERF-VII for proteasomal degradation (Figure 2) [13,15,16]. Conversely, under hypoxic conditions (1–5% O_2_), PCO activity is oxygen-limited, resulting in ERF-VII’s (RAP and HRE) stabilization and subsequent nuclear translocation. Within the nucleus, stabilized ERF-VII proteins activate hypoxia-responsive genes (HRGs), representing a crucial oxygen-sensitive post-translational regulatory mechanism (Figure 2) [2,4,17,18]. It has been reported that both PCOs and ERF-VII mutually regulate their gene expression through fine-tuning feedback. Additionally, ROS signaling pathways (mostly, nitric oxide NO and hydrogen peroxide H_2_O_2_), altered calcium flow, and hormonal mechanisms enhance the response to hypoxia by influencing the expression of HGR genes (Figure 2) [3]. This dynamic interplay enables plants to precisely modulate their physiological responses to the damage generated by hypoxic stress [4,14].

In *Arabidopsis thaliana* there are 5 PCO isoforms (AtPCO 1-5) with distinct expression patterns. While *PCO1* and *PCO2* are hypoxia-inducible, *PCO3*, *PCO4* and *PCO5* maintain constitutive expression [14,19]. Similarly, there are five ERF-VII transcriptions factors described in *A. thaliana*, of which three are constitutively expressed (*RAP2.2*, *RAP2.3* and *RAP2.12*) and two are hypoxia-inducible (*HRE1* and *HRE2*) [16,18,20].

**Figure 3:**
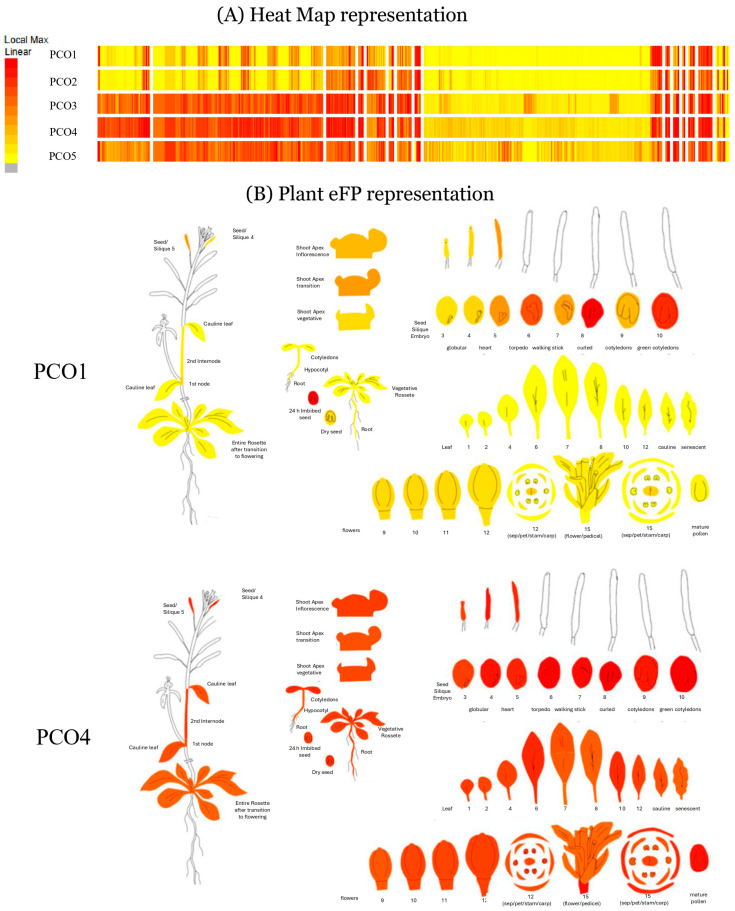
Comparison of relative expression levels (REL) of PCO’s in different tissues of *A. thaliana*. (**A**) Heat Map representation of REL of AtPCO1-5 (**B**) Comparison of REL of PCO1 versus PCO4 in *A. thaliana* tissues. Red and yellow colors represent high and low of REL respectively. This image was generated at https://bar.utoronto.ca/eplant by [24]. The data comes from [22,23].

The direct physical interaction between PCOs and ERF-VIIs constitutes a critical regulatory mechanism enabling plants to respond appropriately to hypoxic stress. For instance, White et al. [14], through kinetic studies, demonstrated that PCO isoforms exhibit distinct substrate specificities toward ERF-VII isoform, with varying catalytic efficiencies. Notably, PCO4 showed the highest activity in oxidizing RAP2.2 and RAP2.12. In contrast, PCO1 and PCO2 displayed comparatively minor contribution to this regulatory mechanism [14]. Furthermore, the interaction between ERF-VII proteins and PCOs may be influenced by specific gene expression patterns. In this context, the presence, absence or differential abundance of proteins in particular tissues can modulate the dynamics of ERF-VII-PCO interactions and determine whether these regulatory complexes are formed or remain inactive. As reported by the BAR eFP Browser at the University of Toronto [21]; PCO3, PCO4 and PCO5 exhibited higher absolute expression levels than PCO1 and PCO2 across multiple tissues, including roots, flowers. hypocotyls, cotyledons, leaves, nodes and rosettes (Figure 3). This pattern suggests a prominent role for constitutively expressed PCOs isoforms in regulating oxygen-sensing pathways in *A. thaliana* [22,23,24].

In addition to the tissue-specific differential expression and the protein–protein affinity between PCOs and ERF-VII, there are biochemical factors that contribute to the plant’s adaptation under hypoxic conditions. For example, White et al. [14] reported that the maximum catalytic activity of the five PCO isoforms in *A. thaliana* occurred within a pH range of 8.0 to 9.0. This activity is lost when the pH in the medium drops to 6.0, which is biologically meaningful, as cytoplasmic acidification occurs in plant cells under anoxic or hypoxic stress. Therefore, it is important that PCOs remain inactive during low O_2_ conditions [14,18,25]. Finally, to respond to hypoxia, ERF-VIIs are regulated through post-translational mechanisms. For example, the constitutively expressed RAP2.2, RAP2.3, and RAP2.12 accumulate in plant cells but remain biologically inactive due to their anchoring to the plasma membrane. Jethva et al. [18] reported that under normoxic conditions, RAP2.12 accumulates at the membrane through its interaction with the acyl-CoA-binding protein (ACBP1). During hypoxia, the ACBP1-RAP2.12 complex dissociates because of decreased ATP levels, allowing RAP2.12 to be released and translocated to the nucleus, where it activates HRGs (Figure 2) [18]. Conversely, *HRE1* and *HRE2* are transcriptionally induced after the detection of hypoxia and play a redundant role alongside RAP2.2, RAP2.3 and RAP2.12 [26,27].

Together, these results suggest a complex regulatory dynamic between PCOs and ERF-VIIs. This complexity arises from the differential and basal expression of the proteins involved, tissue-specific gene expression, and kinetic affinities that respond to fluctuations in the cellular environment, such as changes in pH, cytosolic ion concentrations, the presence of ROS and/or ATP levels. These factors collectively complicate the understanding of the plant’s response to hypoxia.

### 2.2. Role of ERF-VII Transcription Factors During Hypoxia and Plant Immunity

The presence of the N-terminal cysteine (Cys2) as an oxygen sensor in ERF-VII transcription factors in *A. thaliana* suggests a central role in hypoxia regulation (Table 1), besides experimental evidence described below. Studies have shown that RAP2.2 and RAP2.12 are essential for the survival of *A. thaliana* under hypoxic stress, as mutant plants lacking RAP2.2 and/or RAP2.12 exhibit reduced survival rates when low O_2_ condition. In contrast, transgenic lines overexpressing either of these genes display the opposite phenotype, with enhanced tolerance to hypoxia [18,28]. RAP2.3 has a multifaceted role in the response to abiotic stresses, as its overexpression enhances tolerance to heat, hypoxia, osmotic, and oxidative stress [29,30]. Furthermore, plants overexpressing HRE1 or HRE2 exhibit increased tolerance to hypoxia, whereas the double HRE1-HRE2 mutant displays the opposite phenotype, showing heightened susceptibility to hypoxic stress [26,27,31]. Papdi et al. [32] identified RAP2.12 as a transcriptional activator of the alcohol dehydrogenase gene (*ADH1*) observing increased enzymatic activity and high levels of bioluminescence using the luciferase reporter gene (*ADH1-LUC*). The ADH1 enzyme is essential to mitigate hypoxic stress in plants, as it catalyzes the interconversion between aldehydes and alcohols, coupled with the reduction in nicotinamide adenine dinucleotide (NAD^+^) to NADH (Equation (2)). In this context, one of the most significant changes triggered by hypoxia is energy deficit. Under normoxic conditions, ATP is primarily produced through oxidative phosphorylation, which is fueled by glycolysis. However, in the absence of oxygen, ATP is generated via fermentative pathways. In this scenario, the recycling of NAD^+^ to NADH becomes essential for maintaining the proton gradient that drives ATP synthase, thereby helping the plant cope with energy deficit [33,34,35]. Similarly, RAP2.2 plays a critical role in hypoxia survival in *A. thaliana*. Hinz et al. [28] demonstrated that RAP2.2 overexpressing plants (*RAP2.2oe*) exhibited significantly higher survival rates under hypoxia compared to *RAP2.2* knockout lines (*RAP2.2ko*), which showed enhanced sensitivity. Their work further revealed that RAP2.2 partially induces hypoxia-responsive genes, particularly those involved in carbohydrate metabolism and fermentative pathways; modulates ethylene (ET) biosynthesis genes; and participates in ET signaling pathways [28]. Finally, overexpression of *RAP2.3* has been reported to induce the expression of hypoxia marker genes, resulting in an enhanced tolerance to hypoxic stress. This phenotype is proposed to involve ADH1 activation mediated by the hormone abscisic acid (ABA) [30].

A primary alcohol + NAD^+^ = an aldehyde + NADH + H (2)

Equation (2). Reaction catalyzed by the enzyme alcohol dehydrogenase (ADH1). The enzyme ADH1 converts an alcohol into an aldehyde, generating NADH, which is used to generate a proton gradient that drives ATP synthesis.

HRE1 and HRE2 act as hypoxia-responsive transcriptional regulators that sense cellular environment changes during low-oxygen stress. Licausi et al. [26] demonstrated that *A. thaliana* plants overexpressing *HRE1* (*HRE1oe*) showed significantly improved anoxia tolerance compared to wild type. Microarray analysis revealed that *HRE1oe* plants exhibited stronger induction of anaerobic genes under hypoxia than *HRE2oe* plants, which showed no such response. These findings position HRE1 as the primary regulator mediating energy homeostasis through ATP production during hypoxic stress, while HRE2 appears to play a secondary role [26]. However, their results contrast with those reported by Giuntoli et al. [36] who found that HRE1 functions as a repressor of key hypoxia-responsive genes through its physical protein–protein (P-P) interaction with RAP2.12. This interaction enables the plant to readjust its response to hypoxic stress [36]. The apparent discrepancy between the two studies can be explained by two main reasons. First, the formation of the RAP2.12-HRE1 protein complex is highly dynamic and responds to physiological changes that occur under hypoxia, such as the decrease in cytosolic pH and shifts in the cellular redox state. Both factors are known to promote protein–protein interactions and are characteristic of hypoxia conditions. Second, HRE1 has two alternative splicing variants. Seok et al. [37] reported two isoforms, HRE1α and the canonical HRE1β, which differ in length and structural features. HRE1β comprises 262 amino acids whereas HRE1α consist of 211 amino acids. Notably, HRE1α lacks the N-terminal cysteine and an intrinsically disordered region present in HRE1β (Figure 1B). As cysteine residues can form disulfide bonds under oxidizing environments, the absence of Cys2 in HRE1α may limit its interaction capabilities. These isoforms also exhibit distinct functional properties: HRE1β displays stronger transactivation activity than HRE1α, and each variant induces distinct subsets of hypoxia-responsive genes in *A. thaliana* [37,38]. In this context, it is important to investigate the physical, chemical, and/or biological factors present during hypoxic stress that regulate the alternative splicing process of HRE1, as well as the specific conditions that promote the formation of the RAP2.12-HRE1 complex responsible for transcriptional repression.

**Table 1:** Role of ERF-VII during hypoxia and plant immunity.

Transcription Factor	Resistance Hyp	Susceptibility Hyp	Resistance Immunity	Susceptibility Immunity	Reference
RAP2.2	Overexpressed lines *RAP2.2* are Resistant to hypoxia	Lines knockout are sensitive to hypoxia	Overexpressed lines are resistance to *B. cinerea*		[18,26,27,28,39,40,41]
RAP2.3	Overexpressed lines *RAP2.3* are resistant to heat, ROS and hypoxia	Lines knockout are sensitive to hypoxia	Inducible by 1-aminocyclopropane-1-carboxylate (ACC) and methyl jasmonate (MeJA) the same phytohormones involved in the regulation of induced systemic resistance (ISR) and increased expression of *PDF1.2* and *PR-5*		[18,26,27,28,29,30,42,43,44]
RAP2.12	Overexpressed lines *RAP2.12* are resistant to hypoxia			it may act as repressor of jasmonic acid (JA) signaling pathways	[18,26,27,28,32,36,45,46]
HRE1	Overexpressed lines *HRE1* are resistant to hypoxia	HRE1 functions as a repressor by protein–protein interaction with RAP2.12	*HRE1* is inducible by ACC		[18,26,27,28,31,36,38,47]
HRE2	Overexpressed lines *HRE2* are resistant to hypoxia	Double mutant *HRE1-HRE2* are sensitive to hypoxia		*HRE2ko* mutants lines by T-DNA insertion displayed enhanced resistance	[18,26,27,28,31,32,48]

The role of ERF-VII transcription factors during hypoxia is well stablished; however, its relationship with plant defense mechanisms remains unclear. Nonetheless, some studies suggest a potential role in this process. For instance, a study By Zhao et al. [39] demonstrated that RAP2.2 contributes to *A. thaliana* resistance against the necrotrophic pathogen *Botrytis cinerea* (Table 1). Using molecular assays involving overexpressing lines (*RAP2.2oe*) and T-DNA insertion mutants (*RAP2.2ko*), the authors found that *RAP2.2* overexpression enhanced resistance to *B. cinerea*. Moreover, when working with ET-insensitive mutants (*ein2ko* and *ein3ko*) which are lines susceptible to *B. cinerea*, overexpression of *RAP2.2*, restore resistance to *B. cinerea*. These results suggest that RAP2.2 mediates pathogen resistance via ET signaling pathways. In addition, the induction of *RAP2.2* involves the participation of the transcription factors WRKY12 and/or WRKY33, which are necessary for pathogen resistance in *A. thaliana*. WRKY12 and/or WRKY33 can bind to the *RAP2.2* promoter region. Furthermore, through a feedback mechanism, RAP2.2 positively regulates WRKY33 [40,41]. *RAP2.3* has been reported to be inducible by 1-aminocyclopropane-1-carboxylate (ACC) and MeJA, the same phytohormones involved in the regulation of induced systemic resistance (ISR) [41]. Furthermore, transgenic lines expressing *RAP2.3* increased the expression of certain defense genes such as *PDF1.2* and *PR-5* [43,44]. To date, no pathogen challenge experiments have demonstrated direct participation of RAP2.12 in plant defense mechanisms. However, it is known that *RAP2.12* is not inducible by ET and observations suggest that it may act as repressor of JA signaling pathways. Therefore, it is possible that RAP2.12 contributes to hypoxia tolerance through mechanisms independent of ET and maybe also plays a role in increasing pathogen susceptibility by repressing JA-mediated defense responses [26,45,46].

Among the hypoxia-inducible ERF-VII transcription factors, HRE1 and HRE2 have experimental evidence linking them to plant defense responses against pathogens. Recent findings by Yelli et al. [48] suggest that HRE2 is involved in the response to infection by the hemibiotrophic fungus *Fusarium graminearum*. Specifically, HRE2 expression was detected in *A. thaliana* leaves following inoculation with the pathogen. Contrastingly, *HRE2ko* mutants (T-DNA insertion lines), displayed enhanced resistance, as evidenced by smaller lesion areas, reduced cell death and lower levels of fungal DNA compared to wild-type plants (WT). Additionally, repression of the *PDF1.2* gene, which encodes a plant defensin was observed. Collectively, these results indicate that *HRE2* functions as a susceptibility gene, as its expression in WT plants is associated with reduced resistance to *F. graminearum* [48]. In contrast, although there is no direct evidence linking *HRE1* to defense responses against phytopathogens, it has been reported to be inducible by the exogenous application of ACC, an ET precursor [47]. In this context, it is well-established that ET plays a central role in plant immune signaling, contributing to both basal and ISR in coordination with JA [49,50,51,52].

Recently, the dual role of ERF-VII in mitigating hypoxia and responding to pathogen attack has been demonstrated through interaction with the transcription factor ORA59, a key transcription factor for inducing defense genes related to ET and JA hormonal pathways; experimental evidence has shown that ORA59 participates in ET/JA crosstalk through selective recognition of two different cis regulatory elements (the GCC-box and the ERE element). At the same time, it has been shown that ORA 59 modifies the plant’s response to hypoxia by partially repressing the core HGR genes [53,54,55].

Taken together, these findings support the involvement of all five ERF-VIIs transcription factors in mitigating hypoxia in *A. thaliana*. Four of them (RAP2.2, RAP2.3, HRE1, and HRE2) are supported by experimental evidence linking them to resistance against pathogens such as *B. cinerea* and *F. graminearum*. In contrast, RAP2.12 has been proposed to act as a repressor of JA signaling pathways. Furthermore, the physical (P-P) interaction between ERF-VII and plant immunity-related transcription factors such as ORA59, WRKY12, and WRKY33 causes a positive or antagonistic response during pathogen attack.

### 2.3. Regulation of Gene Expression During Hypoxia and Their Role in Defense Mechanisms Through Compensatory Mechanisms

Large-scale genome and translatome analyses in *A. thaliana* have identified a set of 49 core hypoxia-responsive genes (HRGs) that are consistently induced throughout the plant under low-O_2_ conditions [17]. This gene set includes transcription factors, signaling proteins, components of glycolysis and fermentation pathways, as well as genes involved in calcium signaling, ET synthesis, and nitric oxide (NO) metabolism [17,56]. Representative examples among these HRGs include sucrose synthase 4 (*SUS4*; *At3g43190*), alcohol dehydrogenase (*ADH1*; *At1g77120*), and non-symbiotic hemoglobin (*AHB1*; *At2g16060*). While the role of these genes in hypoxia tolerance is well-established, recent studies suggest that they may also contribute to plant defense responses by enhancing the ability to evade or mitigate pathogen attack. For instance, Shi et al. [57] reported that overexpression of *ADH1* confers resistance to both biotic and abiotic stresses, including high salinity, hypoxia, exposure to chemical agents, cold temperatures and enhanced sensitivity to abscisic acid (ABA). ABA is a hormone associated with several physical defense mechanisms, such as callose deposition, which contributes to pathogen resistance [57]. AHB1, a protein with a high affinity for oxygen, plays a key role during hypoxia through its involvement in NO detoxification and/or modulation. Additionally, its expression has been linked to improved resistance against *Pseudomonas syringae* [58,59]. Finally, SUS4 contributes to the reconfiguration of energy metabolism during oxygen deprivation, and its absence has been shown to impair plant’s tolerance to hypoxic conditions [60,61]. Furthermore, SUS4 may also participate in pathogen defense by limiting sugar availability, a mechanism proposed to restrict pathogen access to metabolic resources [62].

The regulation of gene expression extends beyond the interaction between transcription factors and promoter elements; recent findings indicate that O_2_ availability can influence DNA methylation, incorporating an additional layer of epigenetic regulation [63]. Narsai et al. [64] reported significant transcriptomic and DNA methylation changes in rice (*Oryza sativa*) coleoptiles grown under aerobic versus anaerobic conditions, suggesting that the resulting epigenetic landscape differentially regulates cell division and elongation; both of which are physiological responses commonly observed under hypoxia. Similarly, Tsuji et al. [65] observed reversible changes in DNA methylation and histone acetylation in submerged rice plants. Their study reported the transcriptional activation of ADH1 and PDC1 (both hypoxia marker genes) associated with increased recruitment of RNA polymerase type II to their loci. They also detected methylation of Lys4 of histone H3 (Lys4-H3) during early hypoxic exposure (2 h) and increased H3 acetylation during prolonged hypoxia (12 h). These findings suggest that epigenetic mechanisms may play a role in priming and possibly transmitting stress memory across generations. Indeed, Gibbs et al. [63] discussed how enzymatic chromatin remodeling provides long-term hypoxia tolerance mechanisms. This mechanism involves cytosine methylation in DNA and/or nucleosome remodeling. Essentially, chemical modifications (methylation, ubiquitination, and/or acetylation) of histidine tails generate changes in the 3D structure of chromatin, facilitating or disrupting the access of transcription factors to their respective binding sites.

Collectively, these findings highlight the existence of a complex and dynamic regulatory network that modulates plant gene expression under hypoxic stress. They underscore the roles of the *SUS4*, *AHB1*, and *ADH1* genes in mitigating hypoxia and their additional contribution to pathogen defense through compensatory mechanisms. These regulatory processes operate at multiple levels, involving both physical interactions, such as the binding of ERF-VII transcription factors to specific promoter elements, and chemical modifications to chromatin structure, such as DNA methylation and histone acetylation.

### 2.4. Signaling Pathways That Connect Hypoxia with Defense Mechanisms Against Pathogens in Plants

Recent research has identified interconnected between hypoxia and plant defense mechanisms. The induction of a hypoxic state in plants during pathogen infection had been previously reported, the hypoxia affects disease development both negatively and positively by altering the host’s defense response. For example, hypoxia increased the severity of soft rot in potatoes caused by the pathogen *Erwinia carotovora*. In addition, hypoxia increased susceptibility in plants infected with *F. oxysporum*, *Phytophthora cinnamomic*, *Stagonosporopsis tanaceti*, *Fusarium avenaceum* and *Paraphoma vinacea*. Conversely, hypoxia has been shown to have a negative effect on the survival of the fungal pathogens *Collybia fusipes*, *Penicillium griseofulvum*, *Fusarium graminearum* and *Heterobasidion annosum* [63,66]. Subsequently, Tang & Huanhuan [11] mention that hypoxic and pathogen stress occur sequentially or simultaneously in natural environments. As sessile organisms, plants have evolved energy-efficient strategies to cope with this combined stress. The authors described this adaptative mechanism as “single-gene multi-function” where individual genes may serve dual roles in both hypoxia response and pathogen defense [11,67].

Understanding how plants interpret signals to respond to biotic and abiotic stress through a coordinated response has potential applications for the development and improvement of resistant crops [68]. Cell signaling pathways are defined as a group of molecules that work together forming intra- and extracellular communication networks (Signals can be local or distal), activating receptors that allow the cell to integrate the stimulus and determine the cellular response [69]. The signaling pathways during hypoxia and defense mechanism have been extensively studied in plants; oxygen deficiency detection in plants involves multiple sensors and signal transduction pathways. This detection can be direct (by PCO-ERF-VII interaction) or indirect (Homeostasis sensor) and involves an increase in calcium flow by mitochondrial action, modulation of NO triggered by the expression of AHB1 (phytoglobin), ROS signaling, decrease in ATP and hormonal pathways, primarily JA and ABA [3,18,70]. Otherwise, the plant’s defense mechanisms also involve hormonal pathways; however, these depend on the pathogen’s lifestyle. To evade biotrophic pathogens, salicylic acid (SA) signaling is necessary, while for necrotrophic pathogens, JA and ET signaling are required. Additionally, ROS, Ca^2+^, MAP kinases, and protein phosphorylation signaling have been reported [70,71,72].

**Figure 4:**
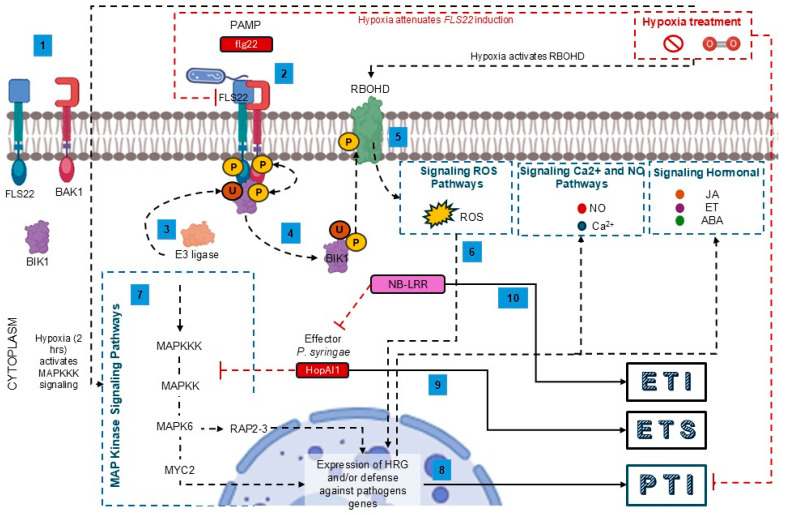
Signaling pathways that connect hypoxia with defense mechanisms against pathogens in plants. (1) Inactive form of the FLS22 receptor (PRR). (2) Interaction between the PRR receptor (FLS22) and the fgl22 (PAMP) and formation of the FLS22-BAK1-BIK1 complex. (3) Ubiquitination of BIK1 by E3 ligase. (4) RBOHD phosphorylation by Bik1. (5) ROS Production by RBOHD. (6) Gene induction and/or repression. (7) The interaction between FLS22 and flg22 induces MAPK signaling. (8) Routes 6 and 7 trigger PTI. (9) The HopAI1 effector of *P. syringae* suppresses PTI by blocking MAPK signaling and triggers ETS (10) The cytoplasmic NB-LRR receptors directly or indirectly identify effectors and trigger ETI. The black dotted arrows signify induction; the red dotted lines signify repression; the blue dotted lines indicate the signaling pathways and the solid black arrows indicate the induction of the defense pathways. This figure was created using BioRender Vega-Arroy & Plascencia-Espinosa (2026) https://app.biorender.com/illustrations/69c49d1a303a83358205571d?slideId=7d521fd8-e0ff-41d6-8c75-b0b40c126409 and Microsoft 365 (Office packages).

The plant’s defense mechanisms are initiated when the transmembrane pattern recognition receptors (PRRs) interact with pathogen-associated molecular patterns (PAMPs; for example, fungal chitin or bacterial flagellin) in response, the plants activate immunity mechanisms; this pathway is known as PAMP-triggered immunity (PTI) (Figure 4). The interaction between PRR’s and PAMP’s generates secondary messengers such as Ca^2+^ and ROS [70,71]. For example, it has been reported that the PRR receptor (FLS2) of *A. thaliana* interacts with bacterial flagellin (effector molecule) specifically with the 22-amino acid peptide fgl22 to trigger PTI. The FLS2 receptor, the BAK1 (transmembrane protein kinase) and BIK1 (a cytoplasmic kinase) protein form a stable protein complex (FLS2-BAK1-BIK1) where transphosphorylation occurs. Subsequently, BIK1 is ubiquitinated by the E3 ligase, releasing BIK1 from the FLS2-BAK1-BIK1 complex to phosphorylate the respiratory burst oxidase homolog D (RBOHD) protein that generates ROS as an essential messenger for the defense of plants against pathogens, highlighting the role of RBOHD in initiating the signaling cascade (Figure 4). Additionally, RBOHD proteins have been shown to actively participate in the induction of hypoxia response genes. Yang & Hong [73] observed the differential expression of *RBOHD* genes in *A. thaliana* under hypoxic stress. Furthermore, they observed that in *RBOHDko* lines, the accumulation of H_2_O_2_ (the main ROS produced by RBOHD) and the expression of *HRE1* and *ADH* genes decreased generating susceptibility to hypoxia [73,74].

Activation of MAPK signaling has been observed following the interaction between FLS2 and fgl22, and this pathway is known to be essential for the activation of PTI. However, some pathogens evade the PTI through effectors (molecules synthesized by microorganisms capable of modifying the plant’s cellular response and promoting infection). Zhang et al. [75] reported that the phytopathogenic bacterium *Pseudomonas syringae* suppresses plant immunity through the HopAI1 effector, which inhibits MAPK signaling. This mechanism is known as effector-triggered susceptibility (ETS) (Figure 4).

In addition to its role in pathogen detection and response, MAPK signaling has been directly linked to the response to hypoxia. The transcription factor RAP2.3 contains a serine residue (Ser151) in its structure, which is phosphorylated by MAPK6, enhancing its DNA-binding activity to GCC-box in in vitro assays and improving its interaction with the transcription factor TGA4; a transcription factor related to defense mechanisms against pathogens suggesting that hypoxia enhances resistance to pathogens through the expression of RAP2.3 and subsequent activation by MAPK6 [75,76,77].

However, these results contrast with those reported by Mooney et al. [78], who reported that hypoxia represses PTI in *A. thaliana*. Using RNA sequencing analysis (RNA-seq), they examined the plant response to fgl22 and identified approximately 4400 differentially expressed genes (DEGs). These DEGs showed overrepresentation in gene ontologies (GOs) such as “innate immune response,” “callose deposition in the cell wall,” and “MAPKKK cascade,” as well as pathways related to oxygen deficiency such as “response to hypoxia” and “regulation of lipid metabolism,” indicating overlap hypoxia and immune signaling.

Then they compared the transcriptional response of Col-0 with that of the *a1a2* mutant (which accumulates ERF-VII and is more tolerant to hypoxia) and founded reduced expression of immunity-related genes including *PDF1.2*, *VSP1*, *CRK25* and *CHIA* in the mutant background. Callose deposition assays further showed that *a1a2* plants displayed reduced callose accumulation upon fgl22 treatment, likely due to decrease expression of *GSL5*, the gene encoding the enzyme glucan synthase-like5 (GSL5), who plays a key role in fgl22 detection. Furthermore, *a1a2* mutants exhibited lower levels of glucosinolates, which are required for callose biosynthesis. Finally, RT-qPCR analysis showed that hypoxia decreases the expression of the PRR genes (*FLS2* and *EFR*). Altogether, these findings indicate that hypoxia represses the transcriptional response of *A. thaliana* to fgl22 peptide through ERF-VII-independent mechanisms and affects PRR levels, callose deposition, and MAPK-associated components [78].

Additionally, there are intracellular NB-LRR receptors (nucleotide binding and leucine rich repeat domains) whose most important function is to recognize effector molecules through direct or indirect mechanisms, triggering effector-triggered immunity (ETI). It has been reported that the SUM protein (NBC-NO) is activated when MAPK signaling is blocked by effectors, culminating in ETI (Figure 4) [71,79].

Taken together, these observations demonstrate an additional level of regulation during hypoxia and plant immunity through the presence of signaling molecules. These stimuli impact the plant’s transcriptional response independently of ERF-VII. Although signaling molecules such as ROS, Ca, and MAPK are functional for different types of stress (biotic and abiotic), the dual involvement of the RBOH10 protein and the phosphorylation of RAP2.3 by MAPK6 significantly enhance the plant’s resistance to hypoxia and its immunity.

### 2.5. Structural Physiological Changes in Plants During Hypoxia and Their Relationship with Defense Mechanisms

The readjustment of gene expression during hypoxic stress induces structural and metabolic changes in plants that may be either beneficial or detrimental to the plant when facing pathogen attack. One of the most prominent changes associated with hypoxia is the alteration of lipid metabolism including synthesis, degradation, and signaling driven by the induction of certain genes such as *LOX1*, *DAD1*, *AOS*, *OPR3*, *and SAD6*, which encode enzymes involved lipid remodeling [78,80,81]. Leon et al. [20] reported that increased unsaturation of ceramides derived from very long-chain fatty acids (VLCFAs) enhances hypoxia tolerance in *A. thaliana* [20]. Consistently, the lipid profile of the hypoxia-resistant *acbp3ko* mutant reveled elevated VLCFA synthesis.

**Figure 5:**
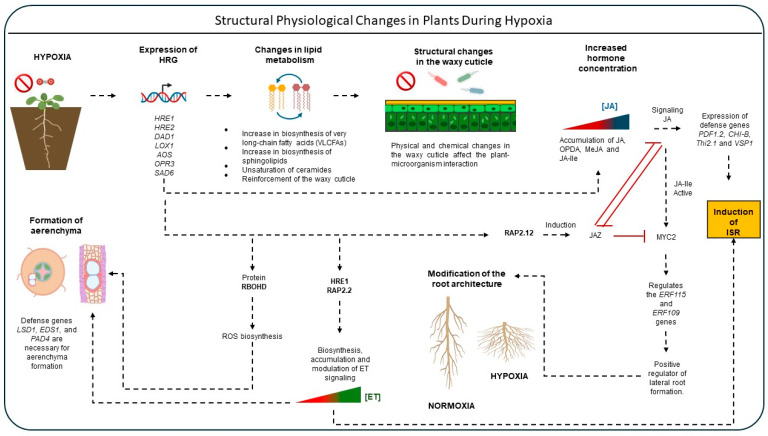
Structural physiological changes in plants during hypoxia and their relationship with defense mechanisms. Hypoxia induces HRGs, which generate lipid readjustment. This lipid readjustment causes structural changes in the waxy cuticle, triggering resistance or susceptibility to pathogens. The induction of HRGs increases the concentration of JA and ET hormones (depending on hypoxic conditions), which in turn leads to pathogen resistance. Simultaneously, these hormones generate structural changes such as the formation of aerenchyma, inhibition of primary root growth, and an increased presence of adventitious roots. The black dotted arrows indicate induction, while the red lines indicate repression; the ramp with a color gradient from red to blue indicates an increase in JA concentration. The ramp with a color gradient from red to green indicates an increase in ET concentration. This figure was created using BioRender Vega-Arroy & Plascencia-Espinosa (2026) https://app.biorender.com/illustrations/69c49ea0303a833582070709?slideId=2889e96f-3837-4ff2-bd32-f9ad9985e18f and Microsoft 365 (Office packages).

VLCFAs (20–40 carbon atoms), synthesized in the endoplasmic reticulum, are essential for multiple biological processes, including defense-related signaling [82,83,84]. They serve as precursors of sphingolipids and of the waxy cuticle. The cuticle, a hydrophobic barrier covering the aerial epidermis, contributes to gas exchange, prevents desiccation, and provides the first physical barrier against pathogen invasion. Its structural integrity is therefore critical for both abiotic and biotic stress tolerance [85]. A well-structured cuticle strengthens plants defenses by limiting pathogen colonization (Figure 5).

In contrast, Kim et al. [86] observed that hypoxia represses the expression of genes associated with cuticular lipid biosynthesis in *A. thaliana*, resulting in increased cuticle permeability and enhanced water influx into the apoplasts. Microscopic analysis revealed structural defects thinner and more translucent cuticles in stems and leaves exposed to hypoxia [86]. Pathogens may exploit these structural changes; for instance, cutin monomers, the primary constituents of the waxy cuticle, can induce extracellular cutinase production in *Fusarium solani* pv. *pisi*, facilitating fungal penetration into host tissues [83,87,88].

Taking together, these results indicate that hypoxia induces lipid readjustment that can be both positive and negative effects on plant defense mechanisms. On the one hand, increased VLCFA and ceramide production strengthens the cuticle and enhances resistance. On the other hypoxia-driven repression of cuticular biosynthetic pathways compromises barrier integrity. These contrasting observations likely reflect differences in experimental conditions including O_2_ concentration (1% vs. 5%), duration of hypoxia, and tissue-specific feedback responses as several studies report opposing phenotypes under prolonged hypoxia. Nonetheless, it is clear that oxygen availability directly influences the physical and chemical properties of the plant’s outer defensive layers, impacting plant-microbe interactions.

Beyond structural modifications, hypoxia-induced lipid reprogramming affects biochemical pathways involved in immunity. One major example is the modulation of oxylipins production secondary metabolites derived from the oxidation of polyunsaturated fatty acids. Key oxylipins include jasmonic acid (JA) and its precursor 12-oxo-phytodienoic acid (OPDA); both of which are enzymatically synthesized and accumulate in response to wounding, herbivory and pathogen attack [49,72]. Shukla et al. [46] demonstrated activation of JA signaling pathways in *A. thaliana* under hypoxia. Chromatographic analysis revealed increased JA, cis-OPDA and jasmonyl-isoleucine (JA-Ile) levels after six hours of oxygen deprivation (from 21 to 1% O_2_). However, these levels declined under prolonged (four days) hypoxia. Complementary, Yuan et al. [89] found that JA and JA-Ile decreased during 24 h hypoxia but increased significantly during reoxygenation, suggesting a role of JA in early hypoxia responses and in the post-hypoxic recovery phase, particularly in mitigating oxidative burst associated with sudden ROS accumulation [89].

JA levels are crucial for inducing ISR and for enabling plants to resist pathogenic microorganisms [49,72]. Mutants defective in JA signaling fail to activate ISR and exhibit increased susceptibility to pathogen infections [89,90]. By contrast, *A. thaliana* mutant plants with constitutively active JA and ET pathways show enhanced resistance, associated with upregulation of defense-related genes such as *PDF1.2*, *CHI-B*, *Thi2.1* and *VSP1* [89]. JA conjugates also mediate long-distance defense signaling: Tamogami et al. [91] demonstrated that MeJA is transported via the vascular system to distal tissues, where it is converted into JA-Ile, which induces the synthesis of volatile organic compounds (VOCs). These VOCs play a role in deterring pathogens and reinforcing systemic defense responses [91,92,93].

Hypoxia also triggers marked changes in root development [78]. Under low-oxygen conditions, *A. thaliana* exhibits inhibition of primary root growth and enhanced lateral root formation, presumably as an energy-saving strategy (Figure 5). Interestingly, this architecture change has also been linked to JA signaling, suggesting that JA regulates not only defense responses but also root developmental reprogramming under hypoxic stress [46,94]. Hypoxia activates the transcription factor RAP2.12 which in turn induces *JAZ* (Jasmonate ZIM domain) genes. JAZ proteins repress JA signaling by inhibiting MYC2 via direct physical interaction. When JA-Ile levels rise, JAZ proteins are degraded via the proteosome, releasing MYC2 and activating downstream genes such as *ERF-115* and *ERF-109*, which modulate root remodeling (Figure 5) [46,95,96].

Finally, the formation of aerenchyma has been observed *in Syzygium kunstleri*, *Gossypium*, *Helianthus annuus*, and *A. thaliana*, among other plants, during hypoxia. These spaces have been shown to serve as oxygen reservoirs and facilitate gas transport (O_2_, ET, CO_2_), primarily in the roots, enabling the plant to mitigate the negative effects of O_2_ deficiency. The formation of this structure is due to ET accumulation and ROS signaling (Figure 5). As previously described, ET plays a significant role in plant immunity; however, the question of whether the formation of this structure (aerenchyma) has any beneficial or antagonistic effect in counteracting pathogens remains largely unexplored. Nevertheless, it has been observed that the defense genes *LSD1*, *EDS1*, and *PAD4* are necessary for aerenchyma formation [97,98,99,100].

In summary, reduced O_2_ availability triggers a lipid readjustment that impacts the synthesis and accumulation of key molecules to defense mechanisms of the plants such as VLCFA, OPDA and JA. The studies discussed suggest that JA acts as a signaling molecule during hypoxic stress, influencing root architecture and defense responses. Notably, the oscillating concentration of JA, MeJA and JA-Ile appear to be time dependent: these compounds accumulate during the early stage of hypoxia (within hours), but their levels decline under prolongate hypoxic conditions (1–4 days). Furthermore, they play a key role during the reoxygenation phase, contributing to the mitigation of oxidative stress and the restoration of cellular homeostasis [46,94]. Finally, the formation of aerenchyma is associated with ET signaling, ROS, and the immunity genes *LSD1*, *EDS1*, and *PAD4*.

### 2.6. Evolutionary Relationship Between Cross-Talk to Hypoxic Stress and Plant Immunity

The convergence of signaling pathways, transcriptional adjustment, and the affinity of ERF-VII for promoter elements present in both HRGs, and immunity genes suggest that cross-talk between the two pathways is the result of a complex evolutionary process. Tang and Huanhuan [11] argue that, in nature, hypoxic stress and biotic stress often occur sequentially or simultaneously; for instance, flooding induces physiological changes that increase plant vulnerability to pathogen colonization. They propose that plants may have evolved to cope with simultaneous stress by inducing multifunctional single genes. Under this scenario, the ability to anticipate pathogen attack while responding to hypoxic stress confer a significant evolutionary advantage compared with strategies in which hypoxia and pathogen responses evolved independently.

Recent research has begun to elucidate the molecular mechanisms underlying this evolutionary convergence. Dalle Carbonare et al. [101] analyzed the transcriptional response to hypoxia across several land plant species and identified both conserved and divergent patterns. Glycolysis and fermentation emerged as universally conserved responses among all species examined. Bryophytes such as *Physcomitrium patens* displayed a relative modest response (98 diferentially expressed genes (DEGs); FDR ≤ 0.01), whereas angiosperms like *A. thaliana* and *O. sativa* showed substancially higher transcriptional reprogramming (1331 and 1952 DEGs respectively; FDR ≤ 0.01). However, the authors noted that direct comparisons are confounded by the intrinsic genetic divergence among taxa. To address this problem, they constructed orthogroups (gene families derived from a single ancestral gene) and identified 4969 orthogroups conserved across clades. Among these, 1543 orthogroups were acquired and 808 were lost in angiosperms, suggesting that the hypoxia response in this lineage diverged significantly from that of bryophites and ferns.

Evolutionary analyses further suggest that ERF-VII transcription factor arose in the last common ancestor of vascular plants with true roots. This origin implies a link between ERF-VII function and the development of vascular tissues and true roots. Genome-wide promoter enrichment analyses revealed an overrepresentation of the GCC-box in *P. patens* (a non-vascular plant). However, algal genomes lack ERF-VII-like proteins as well as ERF proteins containing the conserved Cys2 residue. Taken together, these findings suggest that the interconnection between hypoxia signaling and plant immunity likely emerged latter in plant evolution [101]. Interestingly, the GCC-box was also enriched in genes not responsive to hypoxia, suggesting that the presence of ERF-VII proteins in the nucleus alone is insufficient for promoter binding and impliyng the involvement of an additional, yet unidentified, regulatory components. One such candidate is MBR1 (MED25-binding RING-H2 protein), recently identified as a regulator of the hypoxia response through its interacion with MED25, RAP2.2 and RAP2.12 [67]. The discovery of MBR1 highliths how genetic analyses can reveal new components of PCO-directed proteosomal degradartion pathways [67,101].

In an independent study, Delaux and Schornack [102] analyzed plants and algal genomes and identified conserved genetic modules (groups of co-functional genes) that evolved into increasingly specialized roles across lineages. Their findings emphasize that plant–microorganism interactions have shaped plant diversity throughout evolution. Symbiotic interactions facilitated adaptation to stressful environments, whereas antagonistic interactions with pathogens drove the diversification of plant immune responses. A conserved mechanism across land plants is the detection of PAMPs by PRRs and downstream MAPK signaling [103]. For example, in *P. patens*, the PRR ortholog *Pp*CERK1 activates MPK4a/b, and chitin application induces Ca^2+^ oscillations [75,76,77,100,101,102].

As discussed in Section 2.4, MAPK6-mediated phosphorylation of RAP2.3 enhances the transcription of both HRGs and defense-related genes. However, because *P. patens* lacks ERF-VII proteins, the ERF-VII–GCC-box regulatory module is absent in early land plants.

Taken together, these findings suggest that the integration of hypoxia signaling with plant immunity likely emerged with vascular plants, as bryophytes such as *P. patens* contain GCC-box motifs but lack ERF-VII transcription factors. Nevertheless, we emphasize that additional research is needed to fully understand how hypoxia responses evolved across plant lineages and to clarify whether early non-vascular plants possess alternative mechanisms that may have preceded or paralleled ERF-VII-mediated cross-regulation.

## 3. Future Perspectives

Additional studies are required to verify and expand the knowledge discussed in this review through systematic and controlled experimentation under unified laboratory conditions (model species, duration of hypoxia, and oxygen concentration). Standardizing these variables will be essential for achieving reproducibility and resolving the contradictory phenotypes in plant responses to hypoxia. Although significant progress has been made in understanding the evolution of ERF-VII proteins and their role in hypoxia tolerance [101], future research should determine whether the interconnection between hypoxia signaling and plant immunity arises from compensatory mechanisms or instead reflects a coevolutionary process that integrates environmental cues to simultaneously manage biotic and abiotic stress. Comparative studies in using vascular plants and bryophytes will be crucial to identify conserved or divergent patterns in the response to beneficial and antagonistic microorganisms.

It would be valuable to explore, through functional and comparative genomics, the similarities and differences between the model plant *A. thaliana* and those of agro-industrial crops. As noted by Bailey-Serres et al. [67], environmental factors such as latitude and altitude, as well as the long-term effects of crop domestication, have shaped the evolution of hypoxia responses in plants. For this reason, it is essential to identify both convergent and divergent features of hypoxia signaling between *A. thaliana* and major crops. A striking example of such divergence is highlighted by Loreti and Perata [104], who describe contrasting phenotypic strategies under hypoxia: several rice (*O. sativa*) varieties adopt an “escape” strategy, accelerating stem elongation during submergence due to the activity of SNORKEL genes (*SK1* and *SK2*), whereas *A. thaliana* relies on a “quiescence” strategy, restricting growth to conserve energy. This comparison underscores the importance of studying species-specific and lineage-specific mechanisms to fully understand the evolutionary and functional diversity of hypoxia responses in plants.

A deeper understanding of the interconnection between hypoxia and defense mechanisms will enable the development of crops that are simultaneously tolerant to pests and to hypoxic conditions caused by flooding. Some studies suggest that hypoxia may even present opportunities for crop improvement and food production. For instance, in seeds, hypoxia has been associated with the control of dormancy and germination success, and ethylene signaling under hypoxic conditions can influence fruit ripening in tomato (*Solanum lycopersicum*). These examples highlight that hypoxia responses are highly dependent on spatial and temporal context within specific organs, tissues, or cell types [67,104].

Bailey-Serres et al. [67] emphasize the need to integrate and expand our understanding of the hypoxic response by developing technologies capable of monitoring oxygen concentration in real time across different plant tissues. Such tools would allow researchers to identify and characterize hypoxic niches, which—as discussed previously—can drive distinct transcriptional programs depending on tissue-specific gene expression (Figure 3). Considering this, it is reasonable to propose that hypoxia responses not only differ across plant species but also follow precise spatial and temporal patterns that must be elucidated before this knowledge can be effectively applied to crop improvement.

To this end, the development of biosensors and the application of high-resolution techniques such as single-cell RNA sequencing and immunolocalization will be invaluable. These approaches will facilitate the identification of localized hypoxic microenvironments and the molecular mechanisms through which plants coordinate their responses, ultimately informing strategies for engineering crops better adapted to both abiotic and biotic stress conditions.

A major unresolved question concerns how hypoxia affects plant–microorganism interactions. Because microorganisms differ in lifestyles, hypoxia may either favor or hinder colonization depending on whether the pathogen is necrotrophic, biotrophic, or hemibiotrophic. Specifically, it remains unclear whether the interaction between plants and specific microbes can, in turn, confer increased tolerance to hypoxia. Thus, determining whether the hypoxia–immunity relationship is bidirectional is an important avenue for future research.

Finally, the integration of omics technologies, including transcriptomics, proteomics, metabolomics, epigenomics, phosphoproteomics combined with robust bioinformatics tools, will be critical to generating a comprehensive and integrative understanding on how hypoxia shapes plants defense. Such approaches will help elucidate the fine-tuned regulatory feedback mechanisms that allow plants to adapt their responses to fluctuating environmental conditions and intracellular cues, including pH, ion availability, O_2_ concentration, redox balance, and ATP levels).

## 4. Conclusions

The response to hypoxic stress in plants is complex, exhibiting different levels of regulation (genetic, transcriptional, metabolomic, physiological, etc.). The response can vary depending on the oxygen concentration (0–5%), the exposure time, and/or the induction of feedback signals that allow the plant to readjust its response; this explains the discrepancies in the literature.

Scientific evidence supports the central role of ERF-VII during hypoxia. Furthermore, assays with mutant and/or overexpressing cell lines suggest their involvement in defense mechanisms against pathogens, showing susceptible and resistant phenotypes to infections, as explained in Table 1.

ERF-VIIs can physically bind to the GCC-box promoter region and enhance the induction of defense genes such as *PDF1.2*

It has been experimentally demonstrated that some hypoxia response genes (HRGs) such as *SUS4*, *AHB1* and *ADH1* modify the response to pathogen attack through compensatory mechanisms such as readjustment in NO signaling, increased hormone sensitivity or limiting carbohydrate availability.

There are points of convergence in the signaling pathways activated by hypoxic stress and pathogen stress, highlighting the involvement of relevant proteins such as RBOHD, MAPK6, and RAP2.3 in both cases.

Hypoxia stress generates physiological changes in plants that facilitate or hinder attack by pathogens.

## Data Availability

No new data were created or analyzed in this study. This study is based on previously published data. all data are cited within the article.

## Abbreviations

The following abbreviations are used in this manuscript:

ATP	Adenosine triphosphate
ROS	Reactive oxygen species
DNA	Deoxyribonucleic acid
ERF-VIIs	Members of family VII of ethylene-responsive transcription factors
PCO	Plant protein oxidase
AP2/ERF	APETALA2/ethylene-responsive factor
HRGs	Hypoxia-responsive genes
NAD	Nicotinamide adenine dinucleotide
ADH	Alcohol dehydrogenase
ABA	Abscisic acid
ACC	1-aminocyclopropane-1-carboxylate
MeJA	Methyl jasmonate
ISR	Induced systemic resistance
ET	Ethylene
NO	Nitric oxide
AHB	Non-symbiotic hemoglobin
PRR	Pattern recognition receptors
PTI	PAMP-triggered immunity
PAMP	Pathogen-associated molecular patterns
ETS	Effector-triggered susceptibility
NB-LRR	Nucleotide binding and leucine rich repeat domains
ETI	Effector-triggered immunity
VLCFAs	Very long-chain fatty acids
OPDA	12-oxo-phytodienoic acid
JA	Jasmonic acid
VOCs	Volatile organic compounds
JAZ	Jasmonate ZIM domain
HRE	Hypoxia Responsive ERF
GO	Gene Ontologies
RT-qPCR	Quantitative Reverse Transcription Polymerase Chain Reaction
